# Prognostic value of some inflammatory markers in patients with lymphoma

**DOI:** 10.1042/BSR20182174

**Published:** 2019-03-19

**Authors:** Nahla Hamed Anber, Ahmed H. EL-Sebaie, Noureldien H.E. Darwish, Shaker A. Mousa, Sameh S. Shamaa

**Affiliations:** 1 Fellow of Biochemistry, Emergency Hospital, Mansoura University, Mansoura 35516, Egypt; 2 Hematology Unit, Clinical Pathology Department, Faculty of Medicine, Mansoura University, Mansoura 35516, Egypt; 3 Pharmaceutical Research Institute at Albany College of Pharmacy and Health Sciences, Rensselaer, NY, U.S.A.; 4 Medical Oncology Department/Oncology Center, Mansoura University, Mansoura 35516, Egypt

**Keywords:** chemokines, conventional chemotherapy, Hodgkin, inflammatory cytokines, Lymphoma, non- hodgkin

## Abstract

**Background:** Lymphoma is a group of blood cell tumors which develop from lymphocytes. The main forms of lymphoma are Hodgkin lymphoma (HL) and non-HL (NHL). Cytokines may contribute to lymphoma and they are related to risk NHL and HL. **Aim:** Assessment of the serum level of certain inflammatory markers as complementary indicators to confirm diagnosis of lymphoma patients that may be subjected to more invasive biopsy methods. **Method:** The serum levels of interleukin (IL)-1β (IL-1β), IL-6, IL-10, tumor necrosis factor-α (TNF-α), monocyte chemotactic protein-1 (MCP-1), granulocyte colony-stimulating factor (G-CSF), and eotaxin were assessed by Bio-Plex Pro assays in 81 lymphoma patients and 44 NHL and 37 HL patients before and after chemotherapy treatment as well as 20 healthy persons as a control group. **Results:** Lymphoma patients showed significantly raised marker levels before treatment and significantly reduced levels related to pre-treatment and controls of post-treatment for most of the markers. MCP-1 reported the highest diagnostic accuracy. G-CSF significantly raised pre-treatment and TNF-α. MCP-1 significantly increased in post treated HL compared with NHL. In order to distinguish HL from NHL, G-CSF reported the highest diagnostic accuracy. NHL patients reported complete remission (CR) and those who reported stable disease (SD) and progressive disease (PD) represented 25% and 38% respectively compared with 16% and 27% of HL patients, while partial remission (PR) of HL patients were 56% compared with 36% of NHL patients. **Conclusion:** Most of the markers were significantly increased in pre-treatment but significantly decreased post-treatment. However, it was not considerably enough to get better prognosis of the disease. Elevated serum levels of inflammatory markers correlate with disease severity and low benefit from treatment.

## Introduction

In Egypt, lymphoma represents the most common hematologic malignancy up to 8.4% of all diagnosed cancer types every year. However, the treatment outcome needs to be evaluated and compared with those achieved according to worldwide standards [1]. Lymphomas represent groups of malignant proliferative lymphocytes diseases which originated from T and B cells in the lymphatic system [2]. Lymphomas have been subcategorized into Hodgkin lymphoma (HL) and non-HL (NHL) [3,4].

Development of certain types of cancer, including lymphomas are reported to be stimulated by host immune/cytokine environment. Multiple area of hypoxia is a common feature of solid tumors and many markers are produced in the area of hypoxia such as monocyte chemotactic protein-1 (MCP-1), granulocyte colony-stimulating factor (G-CSF), and eotaxin. These markers are potent chemokines that are chemotactic toward the monocytes in nearby blood vessels [5]. Monocytes cells have been reported to possess dual effects on the tumor, i.e. anti-tumor and pro-tumor effects, with full participation in the angiogenic process [6]. T lymphocytes and monocytes, as inflammatory cells, could control endothelial cell (EC) proliferation as well as their migration and activation [7].

The immune system environment is characterized by high levels of stimulatory cytokines, such as interleukin (IL)-6, tumor necrosis factor-α (TNF-α), and eotaxin produced by nascent tumor cells and/or by reactive tumor-associated immune cells. These markers are expected to contribute to the development of B-cell lymphoma by promoting B-cell activation and associated high-risk DNA-modifying activities [8].

IL-1β is considered to be one of the strong promoting molecules for angiogenesis which stimulates the proliferation of EC, adhesion molecule expression, and production of cytokines and inflammatory molecules that are deeply involved in the angiogenic control process [9]. Along with tumor cells, tumor-associated macrophages (TAMs) produce IL-10, which abrogates the anti-tumor effects mediated by cytotoxic T cells [10,11]. IL-10 induces tumor growth by: direct stimulation of cell proliferation through an autocrine mechanism and by the induction of angiogenesis and the suppression of the local immune system. Suppression of the immune response by inhibiting the T helper cells (Th1) response is considered the main mechanism proposed for the IL-10 to promote tumor growth in humans [12].

Most of the patients with localized disease (stage I or II) and only approximately 30–40% of patients with advanced disease (stage III or IV) were reported to be cured by chemotherapy [13]. Radiotherapy (RT) is considered the first curative approach and almost 70% of patients with localized lymphoma can be cured only by RT [14,15]. In NHL patients, CHOP regimen drug combination (Cyclophosphamide, Doxorubicin, Vincristine, and Prednisone) provides complete remission (CR) rate of 75–80% and also affords range of 3–5-year progression-free survival of 50–80% patients [16]. In HL patients, ABVD chemotherapy regimen combination (Adriamycin, Bleomycin, Vinblastine, and Dacarbazine) is considered the gold standard [17]. A significant number of patients still undergo multiple lines of treatment including high-dose chemotherapy and stem cell transplantation (SCT) with limited outcome due to resistant disease or therapy-associated toxicities [18]. On the other hand, growing insights into the molecular biology of lymphoma has contributed to the development of innovative therapies in recent years. Drugs such as kinase inhibitors are blocking the aberrant B-cell receptor pathways or immune-modulators obtained regulatory approval for treatment of certain NHL entities after promising activity which had been shown in pivotal clinical trials [19].

Elevated serum level of angiogenic and inflammatory markers accompanied with high risk of disease severity and low benefit from conventional adjuvant therapy [20–23].

Biopsy has been considered the gold standard for soft tissue mass diagnosis with a diagnostic accuracy of 94–99%. On the other hand, this invasive procedure has disadvantages—such as being expensive and up to 16% of complication rate (as hematoma), tumor spread, and wound problems—that may interfere with adjuvant treatments. Less invasive methods and other clinical tools are needed to complement routine approaches for diagnosis [24].

Hence, there is need for diagnostic methods with limited risks, high diagnostic accuracy, and faster diagnosis. Inflammatory marker profiles may be used as complementary indicators to confirm clinical and laboratory diagnosis results of lymphoma patients that may be subjected to more invasive biopsy methods depending on their cytokine profile. Patients with lymphoma subjected to histopathological examination of enlarged node(s) to identify type of lymphoma. Also, serum cytokine levels could correlate with disease severity.

For our knowledge, no study has been reported so far to examine the utility of some of the inflammatory cytokines which assisted in serum by quantitating multiple protein biomarkers as prognostic biomarkers panel tool and evaluating the chemotherapeutic-based treatment efficacy in lymphoma. For that reason, the present study was designed to assess serum levels of IL-1β, IL-6, IL-10, TNF-α, MCP-1, G-CSF, and eotaxin by Bio-Plex Pro assays in NHL and HL patients before and after chemotherapeutic-based treatment along with some healthy persons.

## Materials and methods

### Patients

#### Study design

Serum levels of the selected inflammatory markers were assessed by multiplex system Bio-Plex Pro assays in lymphoma patients before and after corresponding chemotherapy treatment along with the serum from a group of healthy persons as controls in a retrospective designee.

Potentially eligible participant patients were 252 cases diagnosed with lymphoma and were selected from outpatient and inpatient at medical oncology units between January 2015 and May 2016 in Oncology Center of Mansoura University, Egypt. Of 252 cases, only 81 cases underwent the present study while 171 cases have been excluded as they suffered other inflammatory disorders or/and other malignant tumors which altered status of the immune and inflammatory cytokines levels that we chose in our study. Patients who were excluded suffered from: hepatocellular carcinoma (*n*=20), chronic hepatitis C (*n*=30), type 1 diabetes (*n*=75), and chronic hepatitis C with diabetes (*n*=46).

Lymphoma diagnosed by clinical examination and radiological investigations for enlarged lymph node was in accordance with the International Working Formulation classification system. According to Ann Arbor staging system with Cotswold modifications—which were based on histopathological examination of biopsy and clinical staging studies—patients diagnosed with lymphoma were classified into NHL and HL of stages II and III [25]. Patients’ ages ranged from 18 to 70 (45.4 ± 13.5) years and were 50 (61.7%) males and 31 (38.3%) females. They included 44 NHL and 37 HL patients. In addition to 20 matched healthy persons aged 18–50 years old were subjected as controls (10 males and 10 females).

#### Clinical significance and chemotherapy treatment schemes

- Patients received chemotherapy treatment according to the assigned treatment protocols. It began with four cycles and were then evaluated. The patients who showed complete remission (CR) had their cycles stopped, while the rest of patients received the completion of chemotherapy to eight cycles.
- NHL patients received CHOP chemotherapy treatment. Scheme of CHOP treatment includes Cyclophosphamide (Cytoxan) 750 mg/m^2^ iv d1, Doxorubicin (Adriamycin) 50 mg/m^2^ iv d1, Vincristine 1.4 mg/m^2^ (max. 2 mg) iv d1, and Prednisone 100 mg po qd d1-5), each cycle for approximately 21 days [26].
- HL patients received ABVD chemotherapy treatment. Scheme of ABVD treatment includes 375 mg/m^2^ iv d1. Each cycle lasted for approximately 28 days [17].
- Response of lymphoma patients to chemotherapy treatment was evaluated as: complete remission (CR); disappearance of all evidence of disease or partial remission (PR); regression of measurable disease and no new sites, or progressive disease (PD); any new lesion or increase by ≥50% of previously involved sites from Nadir stable disease or stable disease (SD); failure to attain CR, PR, or PD [27].
- After treatment, each of NHL and HL patients were subdivided according to their response to selective used chemotherapy regimen treatment into three patients subgroups: subgroup (A), CR; subgroup (B), PR and subgroup (C), SD and PD.
- Pre-treatment levels of the studied markers were compared with those levels after treatment subgrouped and controls. Statistical analyses were established for evaluating those markers role as prognostic tool in diagnosis and also information about therapy regimen efficacy.

#### Ethics approval and consent to participate

Our research has been carried out in accordance with the World Medical Association Declaration of Helsinki and all subjects provided written informed consent. Experiments were approved by the Independent Ethics Committee of the Institutional Review Board of Faculty of Medicine, Mansoura University, Egypt (IRB no. R/17, 11, 75).

## Methods

### Blood sampling

Blood samples were collected before and after treatment of chemotherapy regimen. Samples were allowed to stand for 20–30 min at room temperature to coagulate, followed by centrifugation (2000 rpm, 10 min) and all separated specimens’ serum were immediately frozen and stored in a −70°C freezer.

### Biomarkers assessment

Assessment of serum levels of IL-1β, IL- 6, IL-10, TNF-α, MCP-1, G-CSF, and eotaxin were performed in the Pharmaceutical Research Institute, Albany College of Pharmacy and Health Science, Albany, New York, U.S.A. using Bio-Plex Pro assays. The assessment was performed according to the manufacturers’ instructions: Bio-Plex Pro cytokine, chemokine and growth factor assays for human (Cat# M50007W214, Bio-Rad Laboratories, U.S.A.) which were available in a convenient kit format that included assay, reagent, and diluents components in a single box that quantitated multiple protein biomarkers. Kits included assay reagent(s) and diluents components in a single box. After initial preparation, assay buffer and beads were added to the assay plate. Wash was done by Vacuum Manifold. The filter assay plate was placed on a calibrated vacuum apparatus and the buffer was removed by vacuum filtration. Samples, standards, blank, and controls were added and followed by detection antibodies. Then, strepavidin-PE was added. Plate was read using software protocol which was prepared using normalized standard values. A high-speed digital processor managed data output and Bio-Plex Manager™ 6.0 software was used.

The Bio-Plex® suspension array system instruction was built on the three core elements of xMAP technology:

- Fluorescently dyed microspheres (also called beads), each of those beads have a distinct color code for permitting discrimination of individual tests from a multiplex suspension. This procedure allows simultaneous detection of more than 100 different types of molecules in each well of the 96-well microplate.
- A dedicated flow cytometer with two lasers is associated with optics that permits the detection and assisting the different particles that are bound to the surface of the beads.
- A high-speed digital signal processor manages efficiently the fluorescence data. Data were statistically analyzed using Sigma-Plot program version 10.

### Statistical analysis

Data were fed to the computer and analyzed using IBM SPSS software package version 20.0. Qualitative data were described using number and percent. Quantitative data were described using range (minimum and maximum), mean, S.D., and median. Significance of the obtained results was judged at the 5% level. Mann–Whitney U test was used for non-parametric data to compare two variable quantities. Wilcoxon’s test was used for comparison between parameters before and after treatment and Kruskal–Wallis test used for non-parametric data to compare more than two variable quantities. Receiver Operating Characteristics (ROC) curve analysis identified diagnostic accurate cut-off value which is capable of discriminating between healthy individuals and patients. Validity indices analysis identified diagnostic accuracy, Sensitivity (Sn), Specificity (Sp), Positive predictive rate (PPR), and Negative predictive rate (NPR). Spearman’s correlation was used to correlate amongst parameters. For all statistical analyses, *P*<0.05 was considered statistically significant, *P*<0.01 was highly significant, and *P*<0.001 was considered statistically very highly significant.

## Results

Assessed serum levels of studied inflammatory markers were statistically analyzed in 81 patients diagnosed as lymphoma underwent the present study, 44 NHL and 37 HL patients, and 20 healthy persons as controls. After completion of the chemotherapy treatment regimen, the patients’ groups were classified into four major subgroups; CR, PR, SD and PD according to their response to treatment.

Serum markers’ levels at studied groups are concluded in Table 1. For NHL patients pretreatment, all markers showed significant raised levels except TNF-α, which showed insignificant difference when compared with control. After treatment, all values of the serum markers levels showed significant reduction except IL-6 which recorded insignificant difference when compared with pre-treatment. Eotaxin serum levels reflected significant (*P*<0.05) increase serum level while TNF-α reported significant decrease (*P*<0.05) serum level, in the patients after treatment when compared with control group.

**Table 1:** Inflammatory markers serum levels in NHL and HL patients, before and after treatment, and controls, a comparative analysis study

Markers	Control (*n*=20)	NHL median (range) pg/ml		HL median (range) pg/ml	
		Before treatment (*n*=44)	After treatment (*n*=44)	Before treatment (*n*=37)	After treatment (*n*=37)
IL-1β	1.63 (0.84–2.46)	2.24 (0.69–13.66)	1.43 (0.38–6.04)	2.49 (1.01–6.75)	1.36 (0.55–4.41)
P1		0.008^**^	0.572	0.000^***^	0.851
P2		0.004^**^	-	0.034*	-
P3		0.64	-	-	-
P4		-	0.79	-	-
IL-6	5.65 (1.55–12.4)	10.97 (5.88–119.07)	6.98 (1.15–101.51)	11.77 (6.82–27.7)	6.98 (1.17–26.63)
P1		0.000^***^	0.096	0.000***	0.043^*^
P2		0.13	-	0.028*	-
P3		0.93	-	-	-
P4		-	0.97	-	-
IL-10	5.41 (0.66–10.92)	14.52 (3.31–69.87)	6.93 (1.53–36.8)	13.26 (5.22–30.14)	6.73 (0.66–15.52)
P1		0.000^***^	0.058	0.000^***^	0.062
P2		0.012^*^	-	0.004**	-
P3		0.67	-	-	-
P4		-	0.86	-	-
TNF-α	8.02 (1.59–21.7)	11.53 (3.67–118.73)	5.42 (0.9–17.2)	14.56 (6.81–42.33)	8.33 (0.75–20.59)
P1		0.09	0.033^*^	0.014^*^	0.864
P2		0.000^***^	-	0.003**	-
P3		0.42	-	-	-
P4		-	0.009**	-	-
MCP	4.61 (1.03–10.77)	16.46 (4.79–91.27)	5.46 (1.07–42.29)	17.6 (9.49–51.15)	7.91 (0.78–69.58)
P1		0.000^***^	0.413	0.001^**^	0.001^**^
P2		0.000^***^	-	0.04*	-
P3		0.89	-	-	-
P4		-	0.01*	-	-
G-CSF	36.65(11.28–59.27)	78.09 (12.32–142.1)	44.96 (4.1–138.84)	104.03(37.72–251.65)	43.92 (1.72–148.14)
P1		0.035^*^	0.162	0.000^***^	0.118
P2		0.035^*^	-	0.004**	-
P3		0.014^**^	-	-	-
P4		-	0.81	-	-
Eotaxin	9.195(1.03–31.46)	55.63 (11.27–124.77)	20.86 (2.75–95.43)	43.22 (24.35–145.86)	23.29 (2.03–88.75)
P1		0.000^***^	0.021^*^	0.000^***^	0.003^**^
P2		0.000^***^	-	0.002**	-
P3		0.29	-	-	-
P4		-	0.64	-	-

Abbreviations: P1, versus control; P2, before versus after treatment; P3, NHL versus HL before treatment; P4, NHL versus HL after treatment. * *P*<0.05 **P<0.01 *** *P*<0.001

For HL patients, values of all inflammatory markers assessed read significantly increased levels before treatment when compared with controls. The values of these parameters were significantly reduced in the post-treatment status when compared with those of pre-treatment one. Post treated patients compared with control subject values showed significant increase levels only for IL-6 (*P*<0.05), MCP-1, and Eotaxin (*P*<0.01 each).

Validity measurement for studied serum markers levels were statistically analyzed to identify cut-off value that discriminated between lymphoma patients and control, and detect the diagnostic accuracy of each of those markers. In NHL patients, MCP-1 and eotaxin reported the highest diagnostic accuracy (94.7, 89.5% respectively), Sn (94.4 and 83.3%, respectively), and Sp (95%, each) at serum level >9.075 and >30.88 pg/ml, respectively. Also area under the curve (AUC) for each was 0.97 (95% condense interval (95% CI): 0.91–1.02) and 0.94 (95% CI: 0.87–1.01), respectively. IL-10 and IL-6 showed the second highest diagnostic accuracy (81.6% each), Sn (83.3% each), and Sp (80% each) at serum value >8.37 and >8.11 pg/ml, respectively (Table 2A). For HL patients, the highest diagnostic accuracy was reported for MCP-1 and IL-6 (93.75, 90.63%, respectively) Sn (91.7% each) and Sp (95.05, 90.0%, respectively) at indicated cut-off values >9.38 and >9.59 pg/ml, respectively. Also, MCP-1, eotaxin, G-CSF, and IL-6 identified the same highest sensitivity value (91.7%) and AUC reported values of 0.99, 0.98, 0.95, and 0.93 respectively (Table 2B).

**Table 2:** Inflammatory markers validity measurements of lymphoma patients before treatment

Markers	AUC (95% CI)	*P*-value	Cut-off point (pg/ml)	Sn (%)	Sp (%)	PPV (%)	NPV (%)	Accuracy(%)
**A**: NHL patients								
IL-1β	0.750 (0.59–0.91)	0.009^**^	1.89	72.2	65.0	65.0	72.2	68.4
IL-6	0.881 (0.77–0.98)	0.000^***^	8.105	83.3	80.0	78.9	84.2	81.6
IL-10	0.875 (0.76–0.99)	0.000^***^	8.37	83.3	80.0	78.9	84.2	81.6
TNF-α	0.660 (0.48–0.99)	0.093	9.09	72.2	60.0	61.9	70.6	65.8
MCP-1	0.967 (0.91–1.02)	0.000***	9.075	94.4	95.0	94.4	95.0	94.7
G-CSF	0.700 (0.52–0.88)	0.035^*^	37.0	66.7	55.0	57.1	64.7	60.5
Eotaxin	0.943 (0.87–1.01)	0.000^***^	30.88	83.3	95.0	93.8	86.4	89.5
**B**: HL patients								
IL-1β	0.885 (0.75–1.02)	0.000^***^	2.06	83.3	80.0	71.4	88.9	81.25
IL-6	0.925 (0.83–1.02)	0.000^***^	9.38	91.7	90.0	84.6	94.7	90.63
IL-10	0.887 (0.77–1.00)	0.000^***^	8.16	75.0	80.0	69.2	84.2	78.13
TNF-α	0.762 (0.59–0.93)	0.014^*^	9.045	83.3	60.0	55.6	85.7	68.75
MCP-1	0.992 (0.97–1.01)	0.000^***^	9.59	91.7	95.0	91.7	95.0	93.75
G-CSF	0.950 (0.87–1.03)	0.000^***^	47.96	91.7	85.0	78.6	94.4	87.5
Eotaxin	0.975(0.93–1.02)	0.000^***^	24.88	91.7	85.0	78.6	94.4	87.5

Abbreviations: AUC, area under the curve; PPV, positive predictive value; NPV, negative predictive value; Sn, sensitivity; Sp, specificity; 95% CI, condence interval *P<0.05 **P<0.01 *** P<0.001

Comparative analysis between NHL and HL groups is reported in Table 1. G-CSF serum level at pre-treatment HL group was significantly increased (*P*<0.05) compared with NHL pre-treatment patients group. On the other hand, TNF-α and MCP-1 were significantly increased (*P*<0.01, *P*<0.05 respectively) at post-treatment HL group compared with NHL post treatment patients.

For evaluating efficacy of therapy regimen, post treatment serum markers levels in each of NHL and HL patients’ subgroups were compared with those of controls and those of pre-treatment serum and were statistically analyzed. Compared with controls, markers serum levels at NHL subgroups showed insignificant difference, except IL-10 and eotaxin reported significant (*P*<0.05 each) increase in PR subgroup. Compared with pre-treatment, NHL patients’ subgroups serum levels post-treatment reported significant decrease in median values of IL-1β, TNF-α, MCP-1, and eotaxin (*P*<0.01 each) while IL-6, G-CSF, and IL-10 showed insignificant differences at CR patients. PR patients showed significant decrease in serum levels of IL-1β, IL-6, eotaxin (*P*<0.05 each), TNF-α and MCP-1 (*P*<0.01 each) while IL-10 and G-CSF reported insignificant differences. Also SD and PD patients showed significant decrease in serum levels of IL-10, eotaxin, TNF-α (*P*<0.01 each), and MCP-1 (*P*<0.001) while IL-1β, IL-6, and G-CSF reported insignificant differences (Table 3).

**Table 3:** Inflammatory markers serum levels in NHL patients after treatment subgroups, before treatment and controls, comparative study

Markers	Control (*n*=20)	NHL cases median (range) pg/ml			
		Before treatment (*n*=44)	Subgroup (A) (*n*=11)	Subgroup (B) (*n*=16)	Subgroup (C) (*n*=17)
IL-1β	1.63 (0.84–2.46)	2.24 (0.69–13.66)	1.47 (0.38–2.05)	1.68 (0.5–6.04)	1.55 (0.53–4.23)
P1			0.2	0.7	0.39
P2			0.008^**^	0.045^*^	0.19
IL-6	5.65 (1.55–12.4)	10.97 (5.88–119.07)	7.48(1.67–101.51)	7.1 (2.05–38.84)	9.83 (1.15–42.68)
P1			0.14	0.16	0.08
P2			0.2	0.037^*^	0.19
IL-10	5.41 (0.66–10.92)	14.52 (3.31–9.87)	5.89 (1.53–21.48)	8.99 (1.97–36.8)	6.32 (1.53–36.58)
P1			0.26	0.01^*^	0.36
P2			0.05	0.15	0.006^**^
TNF-α	8.02 (1.59–21.7)	11.53 (3.67–118.73)	4.82 (3.2–12.27)	5.67 (0.9–16.85)	6.29 (1.12–17.2)
P1			0.09	0.09	0.16
P2			0.003**	0.003^**^	0.006^**^
MCP-1	4.61 (1.03–10.77)	16.46 (4.79–91.27)	5.41 (1.65–42.29)	6.73 (1.17–20.07)	5.21 (1.07–13.18)
P1			0.87	0.11	0.56
P2			0.002^**^	0.007^**^	0.000^***^
G-CSF	36.65(11.28–59.27)	78.09 (12.32–142.1)	35.8 (7.56–168.6)	47.53 (4.14–114.2)	50.11(12.68–108.21)
P1			0.56	0.09	0.08
P2			0.4	0.31	0.37
Eotaxin	9.195 (1.03–31.46)	55.63 (11.27–124.77)	20.86 (5.03–55.1)	23.37 (2.03–95.43)	16.89 (2.75–127.15)
P1			0.07	0.03 *	0.18
P2			0.006^**^	0.014^*^	0.002^**^

Abbreviations: P1, versus control; P2, versus before treatment. ^*^P<0.05 ^**^P<0.01 ^***^P<0.001

HL after treatment subgroups serum markers levels compared with controls and pre-treatment levels were reported in Table 4. Compared with controls group, serum markers levels reported insignificant difference in CR patients’ subgroup. Also, insignificant differences for other markers at PR patients’ subgroup were reported except MCP-1 and eotaxin markers serum levels reported significant eleveted levels (*P*<0.05). Significantly elevated levels of MCP-1, eotaxin, IL-10 (*P*<0.01 each), IL-6 and G-CSF (*P*<0.05 each) in SD and PD patients were reported, while IL-1β and TNF-α reported insignificant elevated median levels. Compared with pre-treatment serum levels, CR patients reported significant decrease in all markers levels as, IL-1β, IL-6, IL-10, TNF-α (*P*<0.01 each), MCP-1 (*P*<0.05), G-CSF and eotaxin (*P*<0.001 each); PR patients reported significant decrease levels of all markers serum levels including, IL-1β, IL-6, IL-10, eotaxin (*P*<0.05), TNF-α, MCP-1, and G-CSF (*P*<0.01 each); and SD and PD patients subgroup reported IL-6 and G-CSF, the only markers, showed significant decrease in serum levels (*P*<0.05 each).

**Table 4:** Inflammatory markers serum levels in HL patients after treatment subgroups, before treatment and control, comparative study

Markers	Control (*n*=20)	HL cases median (range) pg/ml			
		Before treatment (*n*=37)	Subgroup (A) (*n*=21)	Subgroup (B) (*n*=10)	Subgroup (C) (*n*=6)
IL-1β	1.63 (0.85–2.46)	2.49 (1.01– 6.75)	1.27 (0.64–4.41)	1.63 (0.55–3.08)	2.04 (1.11–3.65)
P1			0.26	0.98	0.17
P2			0.002^**^	0.027^*^	0.303
IL-6	5.65 (1.55–12.4)	11.77 (6.82–27.7)	5.51 (1.17–26.63)	7.43 (5.21–23.94)	8.5 (6.48–10.83)
P1			0.32	0.059	0.039*
P2			0.005^**^	0.041^*^	0.015^*^
IL-10	5.41 (0.66–10.92)	13.26 (5.22–30.14)	6.65 (0.66–34.51)	6.49 (1.89–15.52)	12.34 (5.74–14.6)
P1			0.33	0.30	0.005^**^
P2			0.005^**^	0.025^*^	0.574
TNF-α	8.02 (1.59–21.7)	14.56 (6.81–42.33)	7.21 (0.75–25.04)	7.69 (4.82–13.65)	11.92 (5.69–13.42)
P1			0.47	0.90	0.25
P2			0.005^**^	0.008^**^	0.261
MCP-1	4.61 (1.03–10.77)	17.6 (9.49–51.15)	6.39 (0.78–69.58)	8.47 (3.15–18.23)	20.19 (5.37–33.75)
P1			0.4	0.01*	0.004^**^
P2			0.01^*^	0.005^**^	0.454
G-CSF	36.65(11.28–59.27)	104.03 (37.72–251.65)	34.60(10.9–148.1)	51.01 (1.72–129.9)	67.82(24.72–87.87)
P1			0.8	0.16	0.015^*^
P2			0.000^***^	0.005^**^	0.015^*^
Eotaxin	9.19 (1.03–31.46)	43.22 (24.35–145.86)	18.6 (2.03–80.19)	28.40 (5.09–88.8)	38.30 (23.3–76.7)
P1			0.11	0.02^*^	0.001^**^
P2			0.000^***^	0.025*	0.606

Abbreviations: P1, versus control, P2, versus before treatment. ^*^P<0.05 ^**^P<0.01 ^***^P<0.001.

## Discussion

Egypt is a country where many epidemic diseases are spread. Here, we studied 252 cases diagnosed with lymphoma, of them 171 cases have been excluded as they suffered other inflammatory disorders or/and other malignant tumors. That was to ensure eligible participant patients with lymphoma alone; however, the number of patients enrolled in the study was more numerous than the calculated sample size. Also, assessed serum levels of studied markers were interpreted without knowledge of the data of the patients. All this was taken into consideration to avoid any source of potential bias or statistical conflict in our work.

NHL and HL constitute heterogeneous group of neoplasm derived from immune system cells. These varieties, as well as the repeated changes over time in lymphoma classification systems, have made it difficult to study their epidemiology [28]. Moreover, understanding the interplay between malignant cells and the tumor microenvironment led to identification of new innovative target therapy in cancer such as kinase inhibitors drugs that block the B-cell receptor pathways or immune modulators drugs such as Lenalidomide for treatment of certain NHL [29].

Inflammatory markers and cytokine levels in serum or plasma are usually assessed by ELISA. It is suggested that biomarker profiles of patients, based on several different biomarkers, could show better diagnostic accuracy than single markers [30].

The highlighted finding of our work was that levels of all serum markers in the pre-treatment status were significantly increased, except TNF-α in NHL which was increased insignificantly, compared with controls. After treatment, some markers were still significantly high and even those showed insignificant differences compared with controls that did not reach the baseline levels. Also, the values of the different serum levels are inflammatory markers after treatment indicated that; although each of NHL and HL patients’ groups has the same diagnosis and the same treatment but they do not have the same response toward the selective medication scheme.

Our results showed that ABND regimen was more effective for treating HL than CHO regimen used for treating NHL, while both these therapies have low benefit for patients. Elevated serum level of angiogenic and inflammatory markers accompanied with high risk of disease recurrent and low benefit from conventional adjuvant therapy [22,23]. Here, the biological variation of tumor was explored to elucidate the potential relevance of our studied inflammatory markers.

Monocytes/macrophages, T lymphocytes, and monocytes are inflammatory cells and fully participate in the angiogenic process [7]. These cells could control EC proliferation, survival, and apoptosis, as well as their migration and activation by secreting pro- and anti-inflammatory molecules such as MCP-1, G-CSF, and eotaxin [5,7]. This agrees with our results that revealed significantly elevated levels of MCP-1, G-CSF, and eotaxin in the lymphoma patients compared with controls and indicating their association with lymphoma disease. Deshmane et al*.* [31] stated that majority of stromal elements, such as macrophages, endothelial, and smooth muscle cells, showed strong cytoplasmic MCP-1 expression which induces chemotaxis of macrophages and lymphoid cells through its receptor chemokine receptor type 2 (CCR2). G-CSF is a glycoprotein molecule that stimulates the mobilization of endothelial progenitor cells (EPCs) into peripheral circulation and promotes angiogenesis [32]. Capoccia et al. [33] reported that G-CSF induces cellular proliferation and migration through direct action on ECs. Eotaxin protein level has been reported directly in correlation with lymphoma and with the extent of tissue eosinophilia. Eotaxin was detected by immunohistochemistry in Reed–Sternberg cell line (RS) cells, fibroblasts, macrophages, and lymphocytes within HL tissues and its expression level is highly elevated than in normal lymphoid tissues [28].

Cytokines play important roles in B-cell activation, proliferation, and apoptosis. Our result showed high levels of IL-10, IL-6, IL-1β, and TNF-α in lymphoma patients compared with control. Our results agree with Skinnider and Mak [34] study which showed the large effects of a wide variety of cytokines and chemokines as IL-6, IL-10, and TNF-α produced by the RS. Also, Elaraj et al. [35] experimental models reported that production of IL-1β influences tumor growth and metastases through direct proliferative effects or by promoting inflammatory and angiogenic pathways in host cells. Skinnider and Mak [34] referred these results to histopathological features of HL which reflect an abnormal immune response composed of mixed inflammatory infiltrate variably that are composed of lymphocytes, eosinophils, fibroblasts, macrophages, and plasma cells. Poor prognosis in many human cancers has been reported to be associated with increased MCP-1 levels and considered as predictor for worse outcome [36]. Also, Gu et al. [37] explained that cytokines may be related to risk of B-cell NHL (B-NHL) by stimulating B-cell proliferation, preventing B-cell apoptosis, and promoting B-cell variable, diversity and joining (B-cell V(D)J) recombination and isotype switching. These reactions are collectively enhancing the chromosome translocations that considered a hallmark of B-NHL. It was reported that the increased risk of B-NHL was associated with variants in the IL-10 and TNF-α genes [38].

The pro-inflammatory cytokine TNF-α plays a central role in immune responses and it is suggested that serum levels are correlated with prognosis in NHL patients [39]. High values of TNF-α serum levels in lymphoma were explained by many studies. It is elevated in NHL patients compared with baseline estimated levels [40]. HL, RS cell line produces high levels of TNF-α that stimulate eotaxin production by fibroblasts [37]. Although TNF-α has anti-apoptotic signals via the nuclear transcription factor (NF)-κB pathway, it influences lymphoma development through pro-inflammatory cytokines such as IL-1β, IL-6, and IL-8 [41,42]. This complemented our results which reflected that IL-1β was highly elevated in lymphoma pre-treatment patient but regretted in the post treatment levels compared with pre-treatment and control levels value.

IL-6 is a key cytokine that promotes the proliferation of hematological malignancies and solid tumors. It is produced by broad variety of cell types including monocytes, fibroblasts, epithelial and ECs as hematological tumor lines [43]. It activates transcription factors STAT through activation of JAK [44]. In NHL, worse progression-free survival and overall survival reports associated with a high IL-6 serum level [45], while production of IL-6 was confirmed in the HL cell lines [46]. Several studies reported that IL-6 expression is coincided with the early onset events of vascular formation suggesting that could play a role in angiogenesis. NHL showed a statistically significantly high value of IL-6 [47]. IL-10 was originally described as a cytokine produced by Th2 cells, B cells, monocytes, macrophages, and keratinocytes. It is known to exhibit both pro- and anti-tumor activities. Moreover, it is reported to be secreted by numerous human cancer cell lines [48]. Some proposed that IL-10 antitumor effect depends on CD8^+^ or CD4^+^ T-cell function [49]. These studies explain and agree with our results because the values of IL-6 and IL-10 levels were found to be higher in patients post treatment and different post-treatment subgroups compared with the control healthy volunteers in HL and NHL reflecting an association between elevated serum levels of those cytokines and aggressive disease and resistance to the used chemotherapy. This is supported by the data from Hong et al. [50] who reported that *in vivo* IL-6, is associated with resistance to cytotoxins and those from Moore et al. [51] who illustrated that IL-10 induces proliferation and differentiation through a potent stimulating effect on B cells. Moreover, serum IL-6 and IL-10 levels were reported higher in NHL and HL patients than in control subjects and correlated with more adverse disease features [52].

Predictive value of chosen markers for diagnosis of lymphoma was identified by validity measurements. MCP-1 reported the highest excellent diagnostic accuracy percentage for NHL and HL (94.7 and 93.75%, respectively) at indicated cut-off levels with a corresponding high sensitivity (94.4 and 91.7%, respectively). In NHL patients’ group, eotaxin, IL-6, IL-10 reported good accuracy values (89.5, 81.6, and 81.6%, respectively) at indicated cut-off levels. For HL disease, IL-6 reported excellent accuracy values (90.6%); G-CSF, eotaxin, IL-1β, and IL-10 reported good accuracy values (87.5, 87.5, 81.25, and 78.13%, respectively) at indicated cut-off value of each marker. IL-1β showed good diagnostic accuracy percentage in HL cases (81.25%) while in NHL cases identified poor value (68.4%). On the other hand, TNF reported the lowest diagnostic accuracy values at NHL and HL lymphoma cases (65.8 and 68.75, respectively) at indicated cut-off values >9.1 and >9.0 pg/ml, respectively. This is agreed with Song et al. [48], who examined the diagnostic value of TNF-α and found low sensitivity and specificity of 59.1 and 57.1%, respectively, with cut-off value of 5.3 pg/ml. Validity analysis results highlighted that, our chosen markers panel tool suggested to provide additional prognostic information superior for NHL and HL disease.

The difference between our work and other studies that our study based on assessment serum level of seven biomarkers simultaneously as a diagnostic panel tool, and our suggestion were supported by Thijs et al. [30] who reported that better diagnostic accuracy is suggested based on several different biomarkers’ profiles of patients than single markers.

Further studies of the diagnostic values of these markers are required before they can be used in clinical practice. When validity of these markers or groups of them are confirmed, biopsy might be no longer necessary and could lead to faster diagnosis and better survival and quality of life.

## Conclusion

High serum levels of inflammatory markers indicated biological aggressive disease with high risk of disease severity and low benefit from conventional adjuvant therapy. Treatment was not considerably enough to get better prognosis of the disease and with low benefit for a group of patients who did not identify CR of lymphoma.

Treatment strategy should be taken into consideration to avoid unnecessary conventional adjuvant therapy in high-risk patients with high serum level of inflammatory cytokines. Also, we found an evidence for the diagnostic utility of the following markers: MCP-1, eotaxin, IL-10 and IL-6 for NHL and HL and IL-1β for HL, while TNF-α has limited utility due to the poor ability to discriminate amongst patients with lymphoma and healthy control, as well as IL-1β for NHL. Accordingly, we can say that studied inflammatory marker may be used as complementary indicators to confirm diagnosis results of lymphoma patients that may be subjected to more invasive biopsy methods depending on their cytokine profile. Also, serum cytokine levels could be used to monitor the severity of the disease and the effectiveness of treatment.

## Abbreviations

ABVD: Adriamycin, Bleomycin, Vinblastine, and Dacarbazine
AUC: area under the curve
B-NHL: B-cell non-Hodgkin lymphoma
CR: complete remission
EC: endothelial cell
G-CSF: granulocyte colony-stimulating factor
HL: Hodgkin lymphoma
IL: interleukin
MCP-1: monocyte chemotactic protein-1
NHL: non-HL
PD: progressive disease
PR: partial remission
RS: Reed–Sternberg cell line
RT: radiotherapy
SD: stable disease
Sn: sensitivity
Sp: specificity
TNF-α: tumor necrosis factor-α
95% CI: 95% condense interval
